# The Madangamines: Synthetic Strategies Toward Architecturally Complex Alkaloids

**DOI:** 10.3390/md23080301

**Published:** 2025-07-28

**Authors:** Valentina Ríos, Cristian Maulen, Claudio Parra, Ben Bradshaw

**Affiliations:** 1 Departamento de Química Orgánica, Facultad de Ciencias Químicas, Universidad de Concepción, Concepción 4070371, Chile; vrios2019@udec.cl (V.R.); cmaulen2018@udec.cl (C.M.); 2 Laboratori de Química Organica, Facultat de Farmacia, IBUB, Universitat de Barcelona, 08028 Barcelona, Spain

**Keywords:** alkaloids, marine sponge, madangamines, *Pachychalina alcaloidifera*, synthesis

## Abstract

Madangamine alkaloids have attracted considerable interest in the scientific community due to their complex polycyclic structures and potent biological activities. The six members identified to date have exhibited diverse and significant cytotoxic activities against various cancer cell lines. Despite their structural complexity, seven total syntheses—covering five of the six members—have been reported to date. These syntheses, involving 28 to 36 steps and global yields ranging from 0.006% to 0.029%, highlight the formidable challenge these compounds present. This review summarizes the key synthetic strategies developed to access critical fragments, including the construction of the ABC diazatricyclic core and the ACE ring systems. Approaches to assembling the ABCD and ABCE tetracyclic frameworks are also discussed. Finally, we highlight the completed total syntheses of madangamines A–E, with a focus on pivotal transformations and strategic innovations that have enabled progress in this field.

## 1. Introduction

**Figure 1:**
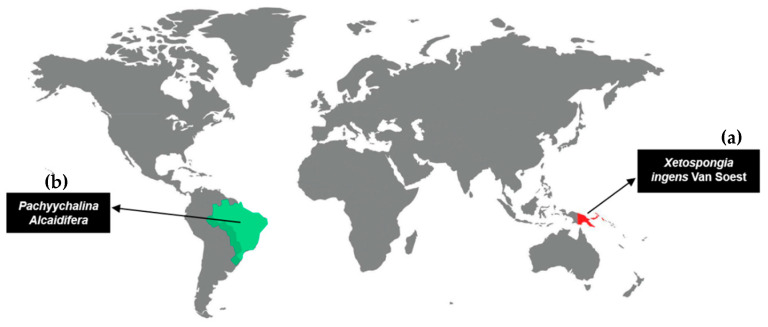
Geographic distribution of marine sponges. (**a**) Papua New Guinea; (**b**) Brazil.

Marine sponges of the order Haplosclerida have been a rich source of marine alkaloids, including complex structures of polycyclic diamines with macrocyclic rings, such as saraines, ingenamines, manzamines, nakadomarine A, and madangamines [1,2,3,4,5,6]. Madangamine A was the first member of a new family of pentacyclic marine alkaloids, isolated by Andersen and coworkers in 1994. This alkaloid was obtained from a marine sponge, *Xestospongia ingens* Van Soest, collected off Madang Province, Papua New Guinea (Figure 1a) [6]. In 1998, Andersen identified four new members of the madangamine family (madangamines B–E) obtained from the same marine sponge source [7]. In 2007, Berlinck identified a new member of the madangamine family from the Brazilian marine sponge *Pachychalina alkaloidifera*, madangamine F (Figure 1b) [8].

**Figure 2:**
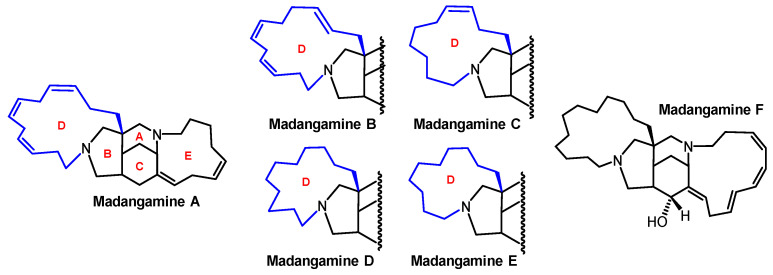
Madangamine alkaloid family.

Structurally, madangamines are pentacyclic alkaloids characterized by a diazatricyclic nucleus (rings A–C) and two macrocyclic rings connecting N-7 to C-9 (ring D) and N-1 to C-3 (ring E) (Figure 2). All madangamines share the same conformation of the diazatricyclic core. However, the peripheral macrocyclic ring D is different in each madangamine, not only in size but also in the degree and position of unsaturation. On the other hand, the E ring is identical in madangamines A–E. Madangamine F, on the other hand, differs structurally from the rest of the family. It contains a longer carbon chain in the E ring and features a hydroxyl group at the C-4 position.

**Scheme 1:**
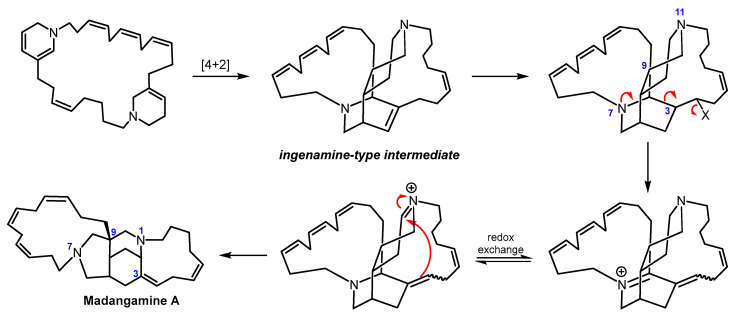
Biosynthesis of madangamine A.

Mandangamine A represents the first example of this class of pentacyclic alkaloids, and its structure has been proposed to originate from the rearrangement of an ingenamine precursor, where the structure undergoes the biological equivalent of a [4 + 2] cycloaddition reaction (Scheme 1). In its biosynthesis, the enzymes responsible for catalyzing this rearrangement may have a specific requirement regarding the chain length and functionality of the C-3 to N-11 bridge in the putative ingenamine precursor. As a result, all generated madangamines have identical N-1 to C-3 bridges but show variations in the N-7 to C-9 bridges. 

The first studies on the biological activity of these natural products were performed on the crude extracts of marine sponges of the order Haplosclerida and Pachychalina alkaloidifera [6]. Andersen isolated 50 mg of madangamine from 200 g of dried sponge, which allowed for its biological activity to be assessed. This alkaloid showed significant in vitro cytotoxicity against several cell lines, including murine leukemia P388 (ED_50_ = 0.93 μg/mL), human lung A549 (ED_50_ = 0.93 μg/mL), brain U373 (ED_50_ = 0.93 μg/mL), and breast MCF-7 (ED_50_ = 5.7 μg/mL) [6]. Based on this background, the cytotoxic activity of madangamine F was also evaluated, showing significant activity against several human cancer cell lines, including SF295 (8.7 μg/mL), breast MDA-MB435 (3.1 μg/mL), colon HCT8 (>25 μg/mL), and leukemia HL60 (6.9 μg/mL) [8]. The results suggest that each of these alkaloids may have a distinct cytotoxic mode of action, likely influenced by the unique three-dimensional structure of each compound. In addition, the relationship between the anti-proliferative activity of madangamine A and its molecular target has been evaluated, identifying this alkaloid as a lysosomotropic agent. These findings demonstrate the potential of madangamine A as a lysosome inhibitor, providing a novel compound for the study of lysosome function [9]. In this context and considering the difficulties associated with the sampling and isolation of these alkaloids, it was of great interest to investigate different routes to obtain them in sufficient quantities to carry out more in-depth cytotoxic studies. This review aims to compile the synthetic efforts made to date to produce these alkaloids, covering both fragment-based approaches and total syntheses.

Despite the complexity of the madangamine alkaloids, seven total syntheses have been reported to date, covering five members of this alkaloid family [10,11,12]. Several studies have been published detailing strategies for constructing the tetracyclic ABCD nucleus [13,14] and synthesizing the diazatricyclic ABC nucleus [15,16,17,18,19,20,21]. The synthesis of an ACE tricycle that includes the macrocyclic E ring has also been reported [22,23]. The following is a summary of the synthetic efforts and strategies employed to date.

## 2. Synthetic Approaches to the Tricyclic Skeleton of Madangamines

### Diazatricyclic Fragment (ABC Rings)

**Scheme 2:**
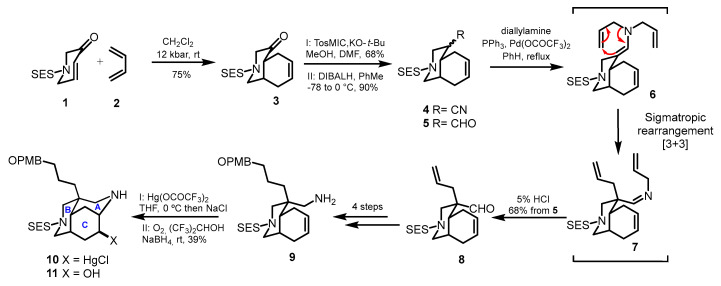
Synthesis of the diazatricyclic ring ABC by Weinreb.

Weinreb and coworkers were the first to propose an approach to constructing the diazatricyclic fragment ABC (Scheme 2) [15]. The cis-fused hydroisoquinoline (BC rings) was first synthesized by a [4 + 2] Diels–Alder cycloaddition of enone **1** with butadiene **2** at high pressure. The conversion of ketone **3** to nitrile **4**, followed by reduction, resulted in an overall one-carbon homologation. To achieve the correct stereochemistry at C-9, aldehyde **5** was treated with diallylamine and Pd(OCOCF_3_)_2_/PPh_3_. The initially generated enamine **6** underwent a stereospecific [3 + 3] sigmatropic rearrangement from the less congested convex face to yield imine **7** which was hydrolyzed to aldehyde **8**. After further manipulation, including a C-9 chain extension to install proper functionality to potentially complete the F ring, the ABC core was completed by an aminomercuration reaction. The treatment of amine **9** with mercury trifluoroacetate to give **10**, followed by exposure to oxygen and NaBH_4_, afforded alcohol **11**, which was suitably prepared for the construction of the macrocyclic ring E, although no further progress was reported [15].

**Scheme 3:**
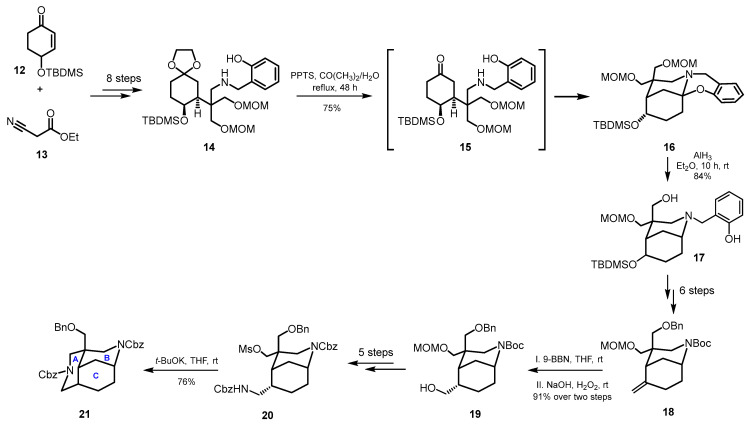
Synthesis of the diazatricyclic ABC ring by Kibayashi.

Kibayashi first prepared the 2-azabicyclo[3.3.1]nonane ring system—featuring the required quaternary carbon at the C-4 position—via N,O-acetalization of a keto-aminophenol (Scheme 3). Starting with the Michael addition of ethyl cyanoacetate **13** to cyclohexenone **12** followed by a further seven steps gave the protected form of **14**. Acetal hydrolysis then gave intermediate **15** which evolved to **16**. Treatment with AlH_3_ resulted in reductive cleavage of the C–O bond of the N,O acetal and selective chelation-controlled deprotection of one of the MOM groups to give **17**. Removal of the (2-hydroxyphenyl)methyl-protecting group by hydrogenation, followed by functional group manipulations, allowed for the required stereochemistry for *cis*-fused ring attachment to be achieved by hydroboration from the less congested convex face of **18** to give **19**. After further manipulations, a base-promoted S_N_2 of the mesylate **20** completed the ABC core **21** in 20 steps. However, the absence of any functional elements for the facile synthesis of the E ring is a limitation of this route [16]. 

**Scheme 4:**
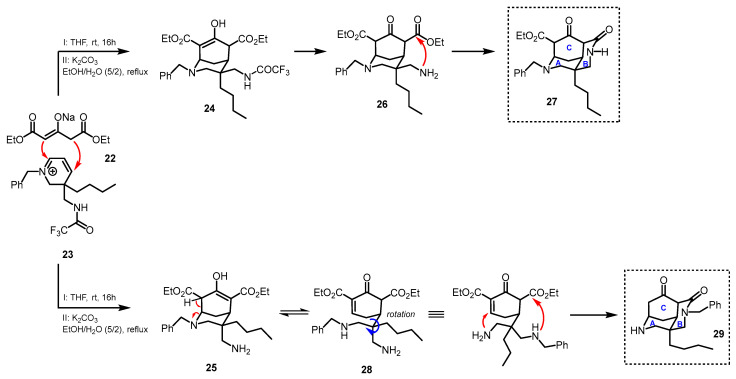
Synthesis of the diazatricyclic ring ABC by Marazano.

Marazano and coworkers adopted an interesting approach based on a biogenetic proposal linking madangamines to ircinals, rather than ingenamine derivatives suggested by Andersen (Scheme 4). Ircinals are related alkaloids found in sponges of the same order. The key step in this pathway was a 3 + 3 nucleophilic addition reaction of the sodium salt of diethylacetone dicarboxylate **22** with a dihydropyridinium salt derivative **23**. In a preliminary study, three adducts were formed; however, after optimizing the reaction conditions, only a mixture of diastereomers **24** and **25** was obtained. Diastereomer **24**, the desired compound from the nucleophilic addition reaction, resulted in diastereomer **27** upon base-promoted cyclization of intermediate **26**. The unexpected formation of compound **29** from the “incorrect” diastereomer **25** arose because the initial adduct **25** is in equilibrium with the enone **28** by a retro Michael process. The rotation of the C−C bond adjacent to the quaternary center then allows for the formation of the amide with the benzylic amino group, followed by the Michael addition of the primary amine and decarboxylation to give the tricyclic derivative **29**. In total, 12 steps were required to access the diazatricyclic nucleus, although the functional groups present limit the possibilities for completing the remaining rings of the alkaloid madangamine [17].

**Scheme 5:**
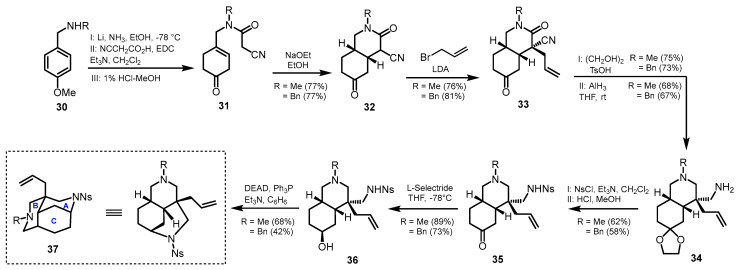
Synthesis of the ABC diazatricyclic ring by Bonjoch.

The synthesis of the ABC tricyclic nucleus by the Bonjoch group was achieved in a 10-step sequence starting from a 4-(aminomethyl)anisole derivative **30** (Scheme 5). The key step in this sequence was the isomerization of the β,γ-enone double-bond **31** induced by a substoichiometric amount of NaOEt and then an intramolecular Michael addition to obtain the *cis* fused B–C ring junction of **32**. The sterically controlled kinetic allylation from the top face introduced the required stereochemistry at the quaternary center to give **33**. Acetalization followed by reduction gave amine **34**. Nosyl protection and acetal deprotection gave **35** which was subsequently stereoselectively reduced by L-Selectride to give alcohol **36**. Finally, a Mitsunobu reaction was employed to complete the diazatricyclic nucleus **37** [18].

**Scheme 6:**
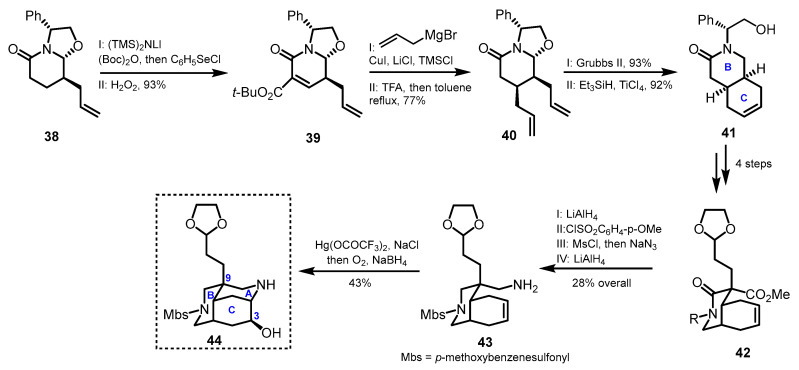
Synthesis of the diazatricyclic ABC ring by Amat and Bosch.

In 2010, Amat and Bosch published the only enantioselective synthesis of the ABC diazatricyclic nucleus of madangamines, which contains the appropriate substitution and functionality for the synthesis of these alkaloids (Scheme 6). The synthesis begins with the enantiopure oxazolopiperidone lactam **38**, which is readily synthesized by a cyclocondensation reaction of (*R*)-phenylglycinol with racemic methyl 4-formyl-6-heptenoate in a process that includes dynamic kinetic resolution. After conversion to the activated unsaturated lactam derivative **39**, a stereoselective conjugate addition of an allyl group followed by decarboxylation gave **40**. Closure of the carbocyclic C ring via an olefin metathesis reaction then generated the *cis*-B/C ring **41**. Subsequently, the quaternary C-9 stereocenter is stereoselectively generated following reductive elimination of the chiral inductor to give **42**. Reduction of the amide and the ester gave an alcohol which was converted to amine **43** in three additional steps. Finally, the ring closure of piperidine A is achieved via an intramolecular aminohydroxylation reaction, exploiting the carbon–carbon double bond of the cyclohexene moiety. This structure features functionality at C-3 and C-9, facilitating the construction of the macrocyclic rings D and E [19].

**Scheme 7:**
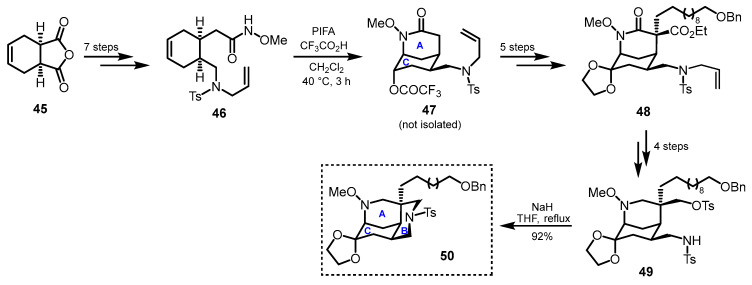
Synthesis of the diazatricyclic ABC ring by Wardrop.

Wardrop synthesized a novel approach to the diazatricyclic madangamine ABC ring system and synthesized a protected advanced intermediate for the synthesis of madangamine D. The synthesis of this nucleus began with a series of manipulations of *cis*-tetrahydrophthalic anhydride **45** leading to the production of the hydroxamate ester **46** in seven steps. Central to the success of this approach is the iodine(III)-mediated intramolecular oxidation of an unsaturated O-methyl hydroxamate **46**, a π−N-type cyclization that proceeds in high yield and with complete regioselectivity to generate the 2-azabicyclo[3.3.1]nonane system **47** spanning rings A and C (Scheme 7). The other key steps in this synthetic sequence were the formation of the quaternary centre to give **48** and the formation of ring B by an intramolecular *N*-alkylation of **49** to afford the diazatricyclic compound **50** [20].

**Scheme 8:**
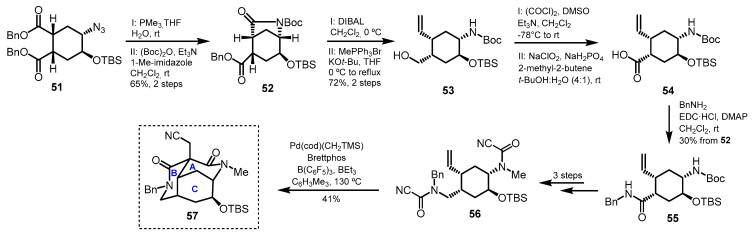
Synthesis of the diazatricyclic ABC ring by Douglas.

The synthesis of the tricyclic ABC nucleus by the Douglas group involves its formation through a cascade reaction. It was carried out starting from diester **51**, which leads to the formation of the bicyclic lactam **52** and thus the introduction of the molecule’s regioselectivity through an intramolecular Staudinger reaction (Scheme 8). After three steps, the monosubstituted alkene and the primary alcohol **53** are formed. The sequential oxidative conditions of Swern and Pinnick gave rise to the carboxylic acid **54**, which, after coupling benzylamine with EDC, leads to the formation of the amine **55**, thereby introducing the second nitrogen. The diamine **56** was obtained after a reduction with LiAlH_4_ and a second protection with *tert*-butyldimethylsilyl triflate (TBSOTf). The key step of this synthesis involves the alkene and the two cyanoformamide groups forming two new rings and a quaternary stereocenter in a cascade reaction, which includes two C–C bond activation steps catalyzed by Pd, leading to the cyanoamidation product **57** [21].

## 3. Synthetic Methods Enabling Macrocyclic Ring Construction

### 3.1. Azatricyclic Fragments (ACE Rings)

**Scheme 9:**
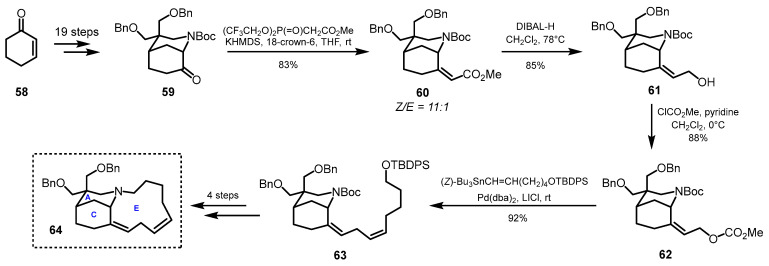
Synthesis of the azabicyclic ACE ring by Kibayashi.

The synthetic route for obtaining the ACE tricycle of the madangamine alkaloids by Kibayashi et al. was similar to that used by the same group but started from the more accessible cyclohexenone **58** (Scheme 9) [22]. This was converted to **59** in a similar manner to that described previously by the same group in Scheme 3. The application of *Z*-selective olefination using the Still–Gennari modification of the Horner–Wadsworth–Emmons reaction, yielded **60**, which was reduced to *Z*-allyl alcohol **61** with DIBAL-H. Palladium-catalyzed coupling of **62** with (*Z*)-vinylstannane gave diene **63** which was isolated as the sole diastereomer. A sequence of deprotection, oxidation, and reductive amination completed the formation of the ACE rings to give **64**.

**Scheme 10:**
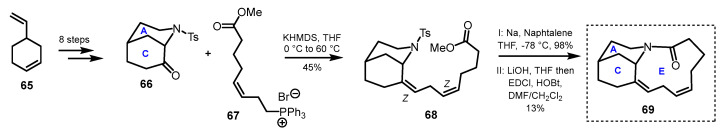
Model for the synthesis of the macrocyclic D-ring of madangamine D by Amat and Bosch.

In their quest to synthesize the macrocyclic rings of madangamines, Amat and Bosch developed various model-based strategies for their preparation [23]. Scheme 10 presents a more direct alternative to Kibayashi’s approach for synthesizing the macrocyclic ring E. This synthesis begins with a series of manipulations of 4-vinyl-1-cyclohexene **65** that lead to an azide and the subsequent formation of azabicyclic ketone **66**, corresponding to the AC rings. Next, a Wittig reaction using the unstabilized phosphonium salt **67** was used to install the required *Z*-alkene adjacent to the 6-membered C ring with similar levels of selectivity to those achieved by Kibayashi (*Z*:*E* = 10:1). Deprotection of the tosyl group and alkaline hydrolysis of the methyl ester of **68** were followed by macrolactamization of the resulting crude amine–acid, yielding **69** as a single diastereomer.

### 3.2. Model Studies on the Construction of Macrocyclic D-Ring

**Scheme 11:**
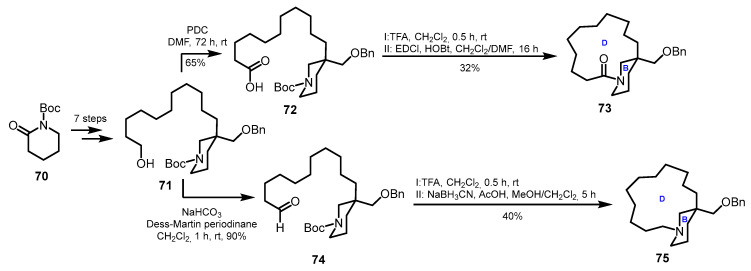
Model for the synthesis of the macrocyclic D ring of madangamine D by Amat and Bosch.

Compound **70** was converted to alcohol **71** in seven steps which was then oxidized to acid **72**. The preparation of the D-ring of madangamine D was achieved using a lactamization strategy to access **73**. Alternatively, oxidation to the aldehyde **74** allowed the D-ring to be closed via a reductive amination to give **75** (Scheme 11).

**Scheme 12:**
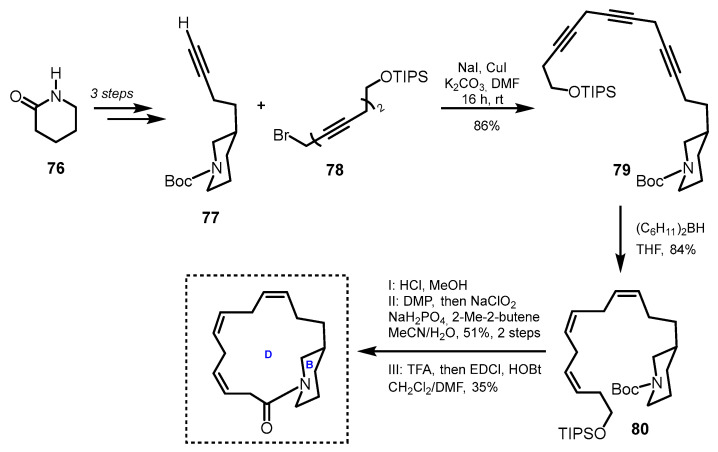
Model for the synthesis of the macrocyclic D ring of madangamine A by Amat and Bosch.

Since the macrocyclic D ring of madangamine A contains three sites of unsaturation, the synthetic approach differs significantly from the previous one [23]. A cuprous iodide-catalyzed coupling of the terminal alkyne of **77** with the diyne **78** gave the triyne chain which was reduced to the non-conjugated triene of ring D using in situ-generated dicyclohexylborane to give the all-*cis*-triene **80** in good yield. Finally, after several standard deprotection and oxidation state adjustments the final annulation step was again performed by macrolactamization using standard coupling reagents (Scheme 12).

**Scheme 13:**
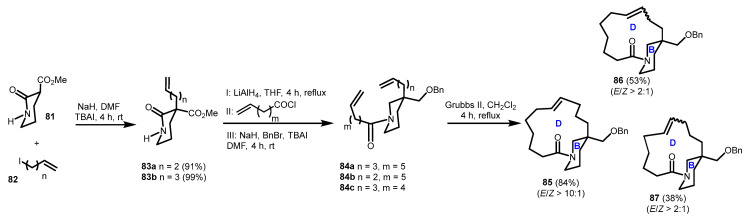
Model for the synthesis of the macrocyclic D ring of madangamines C, D, and E by Amat and Bosch.

Another strategy explored for the construction of the macrocyclic D rings present in madangamines C, D, and E was ring-closing metathesis (Scheme 13) [23]. 3-methoxycarbonyl-2-piperidone **81** was alkylated with unsaturated chains of varying lengths to give dienes **84a**–**c** as detailed in Scheme 13. Ring-closing metathesis of compounds **84a**–**c** yielded the 14-membered lactams **85** and the 13-membered lactams **86** and **87**, respectively.

### 3.3. Tetracyclic Fragments (ABCD and ABCE Rings)

**Scheme 14:**
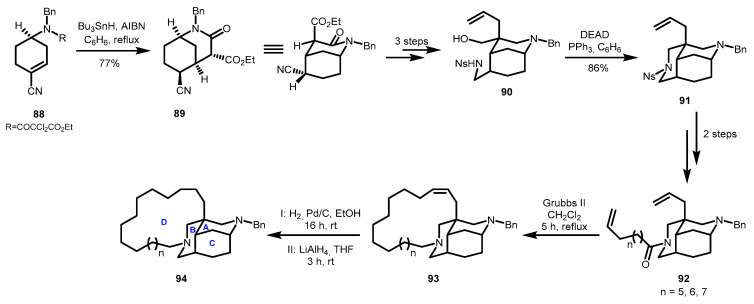
Synthesis of tetracyclic ABCD rings of madangamines D, E, and F by Bonjoch.

Bonjoch’s group completed the first synthesis of the tetracyclic ABCD nuclei of madangamines D–F, starting from a common precursor (Scheme 14). The dichloroamide ester **88** was converted to the morphan nucleus **89** by a reductive radical cyclization with Bu_3_SnH. Subsequently, a chemoselective alkylation, reduction of both the nitrile, ester and the carbonyl group followed by nosylation of the amine leads to the formation of **90**. A Mitsunobu-type aminocyclization of the common diazatricyclic intermediate gave **91** which was then converted to **92** bearing the three different alkyl chains required to construct the different ring sizes of madangamines D–F. Macrocyclization by ring-closing metathesis reaction gave **93** with 13- to 15-membered rings which was subsequently reduced to compound **94 [13]**.

**Scheme 15:**
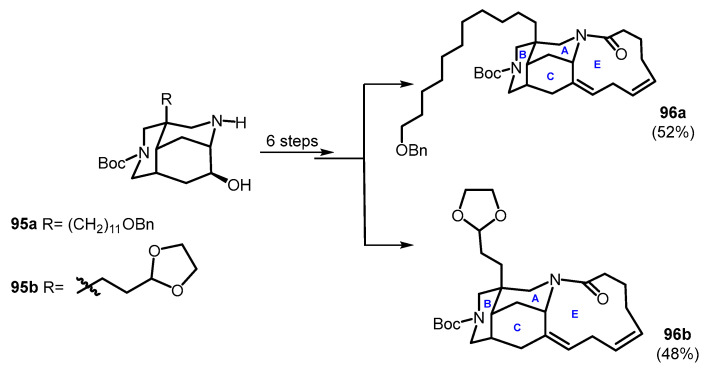
Synthesis of the tetracyclic ABCE nuclei by Amat and Bosch.

In 2019, Amat and Bosch synthesized advanced tetracyclic ABCD and ABCE intermediates of (+)-madangamines A–E, along with a proposal for obtaining the macrocyclic D ring of madangamines B and E (Scheme 15). Initially, the synthesis of the ABCE nucleus was assembled by *N*-tosylation and oxidation of **95a**–**b** to form the corresponding ketones followed by a Wittig reaction. Deprotection followed by alkaline hydrolysis and macrolactamization allows for the formation of ABCE nucleus **96a**, a potential precursor of (+)-madangamine D. On the other hand, under the same conditions, nucleus **96b** is obtained, which has potential in the synthesis of (+)-madangamines A–E [14].

**Scheme 16:**
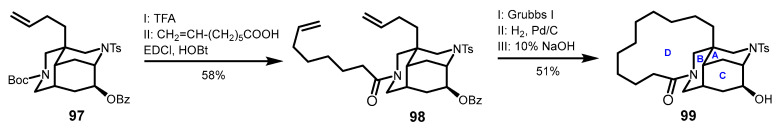
Synthesis of the macrocyclic D ring of madangamine E by Amat and Bosch.

The macrocyclic D ring of madangamine E was obtained from the diazatricyclic ABC nucleus 3-butenyl derivative **97** (Scheme 16). Deprotection of *tert*-butyloxycarbonyl (Boc), followed by acylation with 7-octenoic acid, gives rise to diene **98**, which underwent ring closing metathesis followed by hydrogenation to **99** to construct the macrocyclic D ring resulting in the formation of the tetracyclic ABCD ring system [14].

## 4. Total Syntheses of Madangamines

Despite extensive efforts toward the synthesis of these natural products, as outlined in the previous sections, only three research groups—Bosch–Amat, Sato–Chida, and Dixon—have successfully completed their total synthesis to date.

### 4.1. The First Total Synthesis: Synthesis of the Alkaloid Madandamine D

**Scheme 17:**
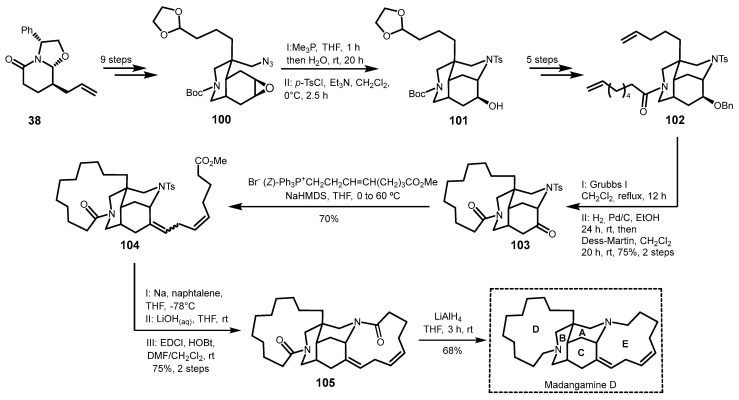
Total synthesis of madangamine D by Amat and Bosch.

In 2014, the Bosch–Amat group reported the total synthesis of madangamine D, the first member of this family to be synthesized [24]. Their pioneering work was highlighted by an efficient ring-formation strategy, which included the rapid construction of the diazatricyclic nucleus, a successful ring-closing metathesis (RCM) reaction to assemble ring D, and the production of ring E by macrolactamization (Scheme 17). In an initial step, a cyclocondensation between (*R*)-phenylglycine and the oxoester occurred, giving rise to the first stereogenic center at C5 of lactam **38**. The following ten steps led to the production of azide **100**, involving key reactions such as a stereoselective conjugate addition, RCM, and stereoselective alkylation and stereoselective epoxidation by m-CPBA. After Staudinger reduction, the generated amine immediately initiated epoxide ring opening to provide diazatricyclic nucleus **101**. The construction of the macrocyclic D ring began with the preparation of dialkene **102** via a three-step sequence: benzylation of the hydroxyl group, selective deprotection of N7, and acylation followed by hydrolysis of the acetal function coupled with a Wittig methylenation. The macrocycle D **103** was then successfully constructed via RCM catalyzed by the first-generation Grubbs catalyst. Finally, the macrocyclic E ring was formed in six additional steps, including deprotection of the hydroxyl group and oxidation to generate a ketone, which enabled the incorporation of an eight-carbon (*Z*,*Z*)-unsaturated fragment via a Wittig reaction to give **104**. Subsequent removal of the tosyl substituent, ester hydrolysis, and macrolactamization afforded compound **105**. A final reduction step with LiAlH_4_ completed the synthesis of madangamine D [24].

### 4.2. Formal Synthesis of Madangamine A

**Scheme 18:**
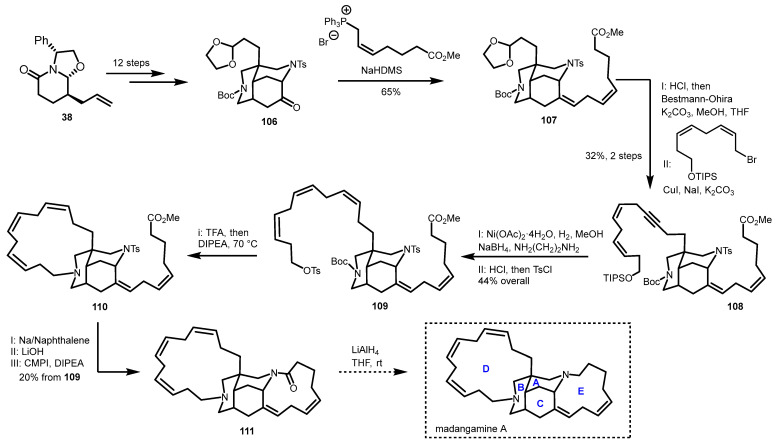
Formal synthesis of madangamine A by Amat and Bosch.

In 2019, the same group also reported the formal synthesis of madangamine A, maintaining the same sequence of steps for the formation of the diazatricyclic ABC ring **106** (Scheme 18). First, ketone **106** allows for the incorporation of the unsaturated chain using a phosphonium salt to give **107**. Hydrolysis of the acetal followed by a Bestmann–Ohira homologation sequence gave a butynyl derivative which, in turn, reacts with *Z*,*Z*-octadienyl bromide in a CuI-catalyzed cross-coupling to furnish dienyne **108**. Next, the stereoselective reduction of the resulting diene was carried out giving rise to *Z*,*Z*,*Z*-triene moiety required for the D ring of madangamine A. Exchange of the TIPS-protecting group for a tosyl group gave **109** which after deprotection of the *N*-Boc group undergoes intramolecular alkylation to give **110**. Deprotection of the *N*-tosyl group, alkaline hydrolysis, and macrolactamization was then undertaken for construction of the macrocyclic E ring to give **111** which constituted a formal synthesis of madangamine A [25].

### 4.3. Divergent Strategy: Total Synthesis of Madangamines A, B, C, D, and E

**Scheme 19:**
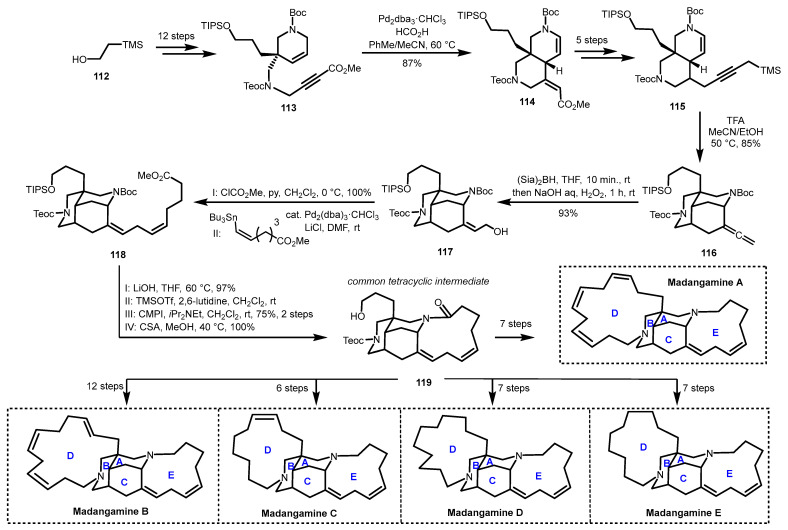
Synthesis of madangamines A–E by Sato and Chida.

The identical E ring in all madangamines A–E inspired the Sato and Chida group to devise an advanced intermediate for the divergent synthesis, constituting the total syntheses of madangamines A–E (Scheme 19). In 2017, Sato and Chida performed a new unified total synthesis, which, like the previous proposal for synthesizing the madangamine alkaloids A, C, and E, was based on forming a common ABCE tetracyclic intermediate. This approach gave them the appropriate conformation to proceed with the formation of the D ring. Obtaining the common intermediate begins with converting 2-(trimethylsilyl)-ethanol **112** to **113** in 12 steps. In this sequence the key steps involve a nickel-catalyzed [4 + 2] cycloaddition, an anti-S_N_2 under Kobayashi conditions, a Birch reduction, and carbonylation of the terminal alkyne. Methyl alkynoate **113** then gives way to the formation of ring B by a palladium-catalyzed isomerization cycle furnishing **114**. Five subsequent steps involving aldehyde formation and Bestmann–Ohira reaction homolgation and alkylation with (iodomethyl)trimethylsilane allowed for the production of propargylsilane **115**. Protonation with CF_3_COOH (TFA) resulted in cyclization, giving rise to a 1,1-disubstituted allene **116**. The next step involves the construction of the (*Z*,*Z*)-skipped diene, which was carried out by selective hydroboration, where (Sia)_2_BH allowed for the production of the allylic alcohol **117** (*E:Z* = 1:20). After the conversion of the alcohol into a methyl carbonate, a coupling reaction generated the alternating (*Z*,*Z*)-diene **118**. Along with several functional group manipulations, cyclization generates the macrocyclic E ring and gives rise to **119** [11]. From this common intermediate, the construction of the macrocyclic D ring proceeded through a different number of steps depending on the target alkaloid. The side chain was introduced via a Wittig or Grignard reaction in the case of madangamines A, C, D, and E followed by ring closure. The synthesis of madangamine B was particularly challenging due to the unique positioning of the double bonds in the D ring. This complexity necessitated shortening the carbon chain of intermediate **119** and introducing the remaining carbon atoms through two distinct processes. As a result, this final portion of the synthesis became the longest sequence, requiring twelve steps to complete.

### 4.4. Enantioselective Total Synthesis of Madangamine E

**Scheme 20:**
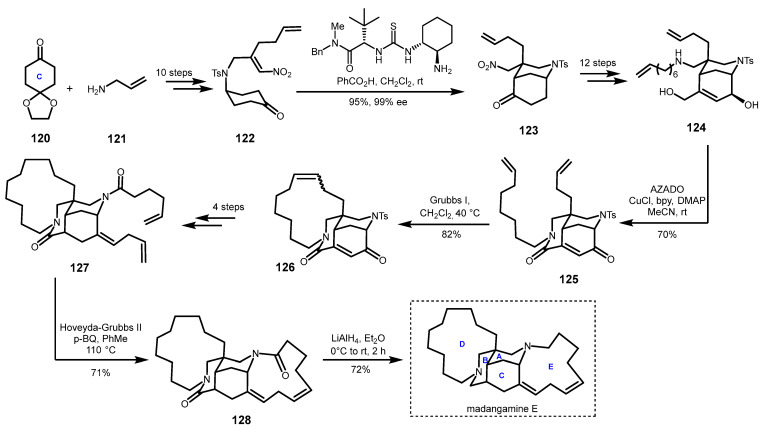
Total synthesis of madangamine E by Dixon.

The total synthesis of madangamine E by the Dixon group in 2022 involved several key reactions that were crucial for constructing the complex structure of this alkaloid. Notably, an enantioselective desymmetrization facilitated by an organocatalyst enabled access to the morphanic A–C ring system (Scheme 20) [12]. Initially, a nine-step sequence was applied, starting from acetal **120** and allylamine **121** to obtain nitroolefin **122**. Then, the asymmetric Michael addition was developed using a thiourea catalyst, allowing for the formation of bicyclic intermediate **123** in 95% yield and 99% enantioselectivity (ee). After 12 steps they obtained allylic alcohol **124** which was selectively transformed into lactam **125** by the Iwabuchi oxidation. The cascade process likely involves the hemiaminal as an intermediate, which is further oxidized to release the desired lactam. The subsequent RCM reaction using the Grubbs I catalyst allowed for the formation of ring D of compound **126** in an 82% yield. Chain elongation at C3, along with installation of the alkene chain on the nitrogen atom, produced **127**, which was primed for the formation of ring E via a second ring-closing metathesis (RCM) using the Hoveyda–Grubbs II catalyst to give **128**. Finally, the resulting pentacyclic compound was readily converted to madangamine E by reduction of the two amide groups with LiAlH_4_.

## 5. Conclusions

This review summarizes the major achievements and challenges in the synthesis of madangamine alkaloids, providing a comprehensive overview of what has been reported to date [26,27,28]. These compounds, isolated from marine sponges, are of great interest due to their complex polycyclic structures and their potent cytotoxic activities against various cancer cell lines. The structural complexity inherent in their diazatricyclic (ABC) and macrocyclic (D and E) nuclei has driven the development of ingenious synthetic strategies by multiple research groups. We summarized the various approaches for constructing the ABC and ACE fragments and the ABCD and ABCE tetracyclic systems, highlighting the diversity of methods employed to address these structural challenges. Pioneering total syntheses, particularly that of madangamine D by Bosch–Amat and the enantioselective syntheses of madangamines A–E by the Sato and Chida group, represent crucial milestones that have allowed broader access to these compounds. Despite these advances, the difficulty in sampling and isolating madangamine alkaloids underscores the ongoing need for efficient, scalable synthetic routes. Future research could focus on developing new, more concise routes based on function-oriented synthesis [29], which could reduce the synthetic challenges posed by these alkaloids and potentially lead to the discovery of new therapeutic agents.

## Data Availability

Not applicable.
